# Evaluation of the implementation progress through key performance indicators in a new multimorbidity patient-centered care model in Chile

**DOI:** 10.1186/s12913-023-09412-9

**Published:** 2023-05-04

**Authors:** Teresita Varela, Paula Zamorano, Paulina Muñoz, Carolina Rain, Esteban Irazoqui, Jaime C. Sapag, Alvaro Tellez

**Affiliations:** 1 Centro de Innovación en Salud ANCORA UC, Facultad de Medicina, Pontificia Universidad Católica de Chile, Santiago, Chile; 2 Health Technology Assessment Unit, Center of Clinical Research, Pontificia Universidad Católica de Chile, Santiago, Chile; 3 Current Address: Diagonal Paraguay, Santiago, 362 Chile; 4 Department of Family Medicine, Pontificia Universidad Católica de Chile, Santiago, Chile; 5 Department of Public health, Pontificia Universidad Católica de Chile, Santiago, Chile; 6 Dalla Lana School of Public Health, University of Toronto, Toronto, Canada

**Keywords:** Multimorbidity, Key performance indicators, Implementation, Progress, Patient-centered, Model

## Abstract

**Background:**

Complex health interventions involve deep organizational, structural, and cultural changes that challenge health teams and decision-makers. The explosion of chronic diseases has made the multimorbidity approach a global priority. The Centro de Innovación en Salud ANCORA UC implemented a Multimorbidity Patient-Centered Care Model in the Chilean public health system.

**Objective:**

This study aims to evaluate the progress of the implementation of the Multimorbidity Patient-Centered Care Model in seven primary care centers through key performance indicators.

**Methods:**

a set of indicators was designed to evaluate change management, operations, installation of new roles, and services and activities of the intervention strategy of the model. Key performance indicators were identified to monitor the implementation progress on minimal components for the model’s sustainability. Each item was assigned against an expected minimum score of 67% of progress from the overall score. They were monitored twice in seven primary health centers in 2019 and 2020, which intervened 22,642 patients with the intervention.

**Results:**

The results showed that six of the seven primary care centers reached the minimum implementation threshold. The main advances were in operational conditions, and those with minor progress in implementation were the clinical services. Population size, organization, coordination of the health care teams, additional training, and decision-makers support were key factors that determined the degree of progress in a complex intervention.

**Conclusion:**

It was possible to measure the progression of the implementation of a complex intervention through key performance indicators delivering relevant information for decision-makers that pursue a successful and faithful implementation. This study provides a valuable tool for the national scale-up of a similar model started in Chile by the Ministry of Health and other countries.

**Supplementary Information:**

The online version contains supplementary material available at 10.1186/s12913-023-09412-9.

## Introduction

Complex changes in health represent a real challenge for health systems, clinical teams, and individuals not only because of their inherent complexity but also in terms of ensuring sustainability over time. For example, in recent years, important epidemiological changes have modified the burden of disease, health services use, and life expectancy, making the reorganization of health services a priority [1]. Therefore, complex changes require core elements that allow change to be executed and its sustainability, such as changes in associated resources, adequate competencies, clear leadership, and culture and behaviors that support change [2].

Methodologies and frameworks for implementing complex changes in health described usually include stages of theory exploration, development of clinical intervention, identifying core aspects, as well as feasibility and implementation studies [3]. The evaluation is often focused on intermediate or final outcomes, but they lack performance indicators that can deliver valuable information from the implementation process. Even more, challenges of the complex interventions are born within the implementation process, from operational and leading changes barriers that need to be appropriately addressed to pursue a sustainable incorporation [4].

Measurements during complex interventions are core in monitoring the degree of implementation progress of the proposed intervention. A healthcare Key Performance Indicator (KPI) is a clear-cut measure used to observe, monitor, optimize, manage, and transform the performance of a healthcare process to ensure effectiveness, quality, and efficiency and increase patient satisfaction and healthcare providers [5, 6]. Therefore, their use in complex interventions could provide a broader perspective with quantitative and qualitative information that can help decision-makers during the process. For example, when a complex intervention involves organizational, operational, and cultural changes, KPIs can monitor/track progress and make objective comparisons between different contexts enabling opportune response to those who are experiencing a harder process [7]. However, according to the quality and amount of health services data, it is key to choose the indicators that can be fulfilled simply and provide relevant information to the process and progress of the implementation of a complex intervention.

**Fig. 1:**
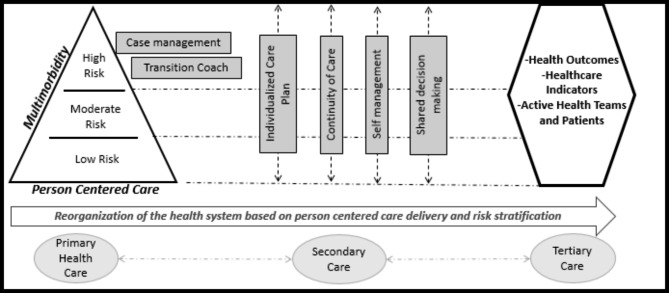
Multimorbidity Patient-Centered Care Model *(MPCM)* [12]

There are experiences in Chile and internationally that show how complex the profound changes to the organization and delivery of health services are. In the field of multimorbidity defined as two or more chronic conditions in the same person [8], the Chilean public health system and its primary care centers are organized in the traditional single diagnostic approach. They are offering fragmented disintegrated, and inefficient care, which has shown negative results in 11 million people (70% of the national population) living with chronic disease outcomes during the last years [9, 10]. Therefore, the Centro de Innovacion en Salud ANCORA UC (CISAUC), together with the Servicio Metropolitano Sur Oriente (SSMSO) and National Found of Health (FONASA), implemented a complex change in health. The objective was to change health services organized in diagnosis towards a patient centered care organized according to each patient multimorbidity risk. The Multimorbidity Patient-Centered Care Model (MPCM) enhances the family and community health model implemented in the primary health care centers (PHC) of the country [11] and adds core elements such as case management, risk stratification, and multimorbidity as shown in Fig. 1. The intervention strategies were designed and offered in primary and tertiary care centers, according to each person’s risk.

**Fig. 2:**
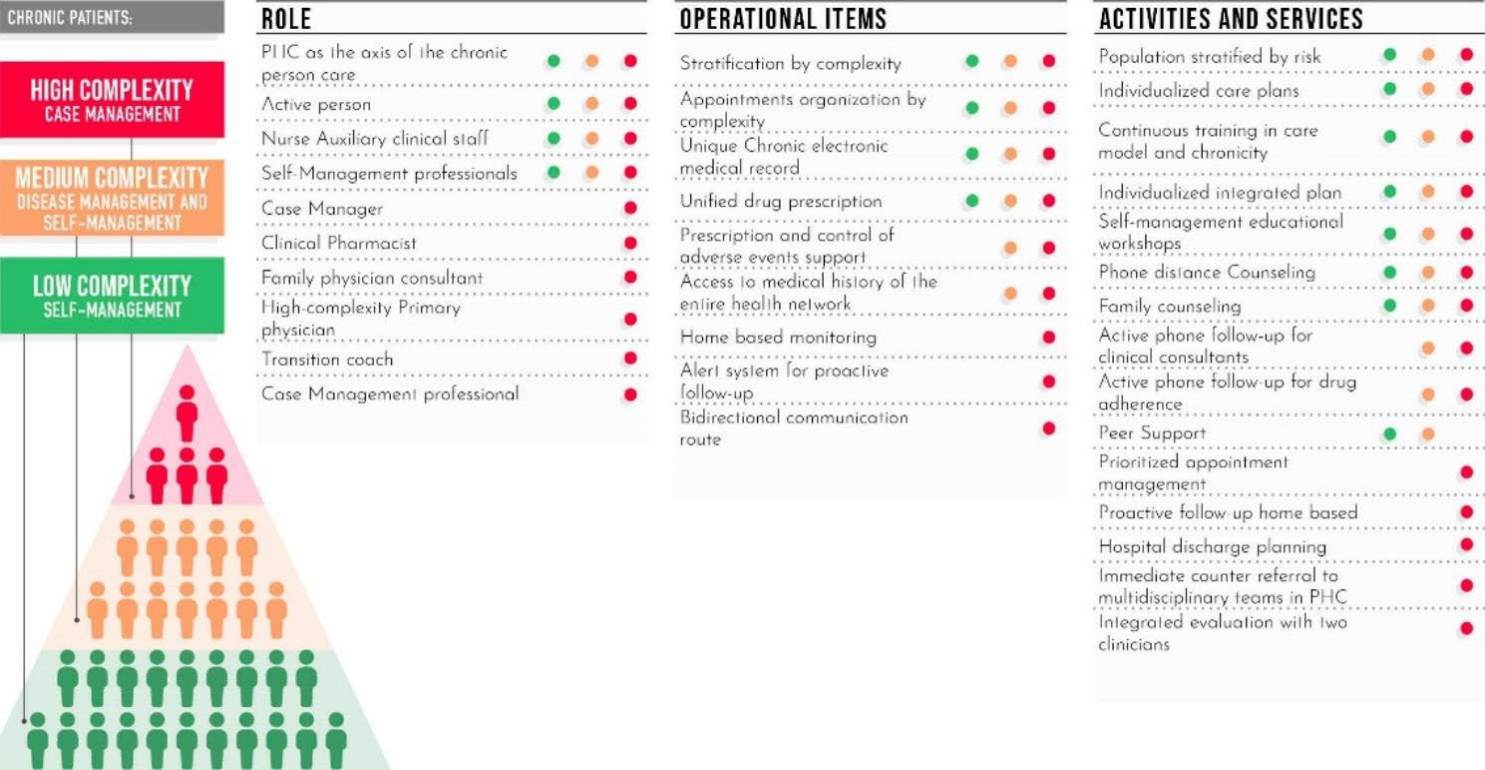
Intervention strategy of the MPCM *Adapted from Care Strategy for patients with chronic diseases in the community of Madrid, November 2013, Ministry of Health, Social Services and Equality* [14].

The implementation process had three stages: preparation, implementation, and evaluation activities. During the preparation, process activities were carried out to disseminate and communicate the model, together with training of health teams and operational preparation. In the implementation, clinical activities corresponding to the intervention strategy were executed (Fig. 2), and the CISAUC expert team monitored each center’s implementation’s particularities and execution times. In the evaluation stage, an impact analysis on the use of health services and an evaluation of patient and health team satisfaction were carried out showing positive results [12]. Still, measuring the impact on avoidable hospitalization would have complemented those results. Similar interventions have shown a decrease in unplanned hospitalizations [13]. The MPCM intervention decreased the total number of hospitalizations, and we could infer that those results are related to the decrease in avoidable hospitalization. But at the time of evaluation, there was a lack of consensus about the kind or list of avoidable hospitalizations, limiting the data extraction and evaluation. Given that there were multiple barriers and facilitators that influence the progress of implementation and its sustainability over time, this study aimed to evaluate the progress in implementing the Multimorbidity patient-center care model in seven Primary Care Centers in Chile.

The study’s objective was to evaluate the progress of implementing the Multimorbidity Patient-Centered Care Model in seven primary care centers through key performance indicators.

## Methodology

**Fig. 3:**
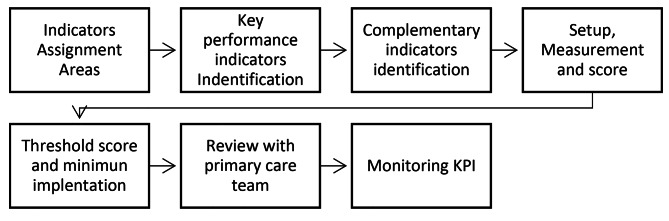
Key performance indicators setup, scoring and monitoring process

The study used a quantitative approach to assess the progress of implementing the MPCM in seven primary health care centers in the southeast of Santiago, Chile, that intervened in 22,642 adult patients with multimorbidity. The PHCs are organized by the Family and community model [15]. Their size ranged from 3 to 4 multidisciplinary health teams to offer care from 22,000 to 35,000 patients (covered population) with vulnerable conditions. The intervention strategy shown above (Fig. 2) had several components from which developed a set of indicators in four main areas: change management, operational items, new roles, and services and activities. In addition, some KPIs were identified from the overall set of indicators to reflect the minimum conditions required for the intervention sustainability. Figure 3 shows the process of the setup and monitoring of KPI.

### Indicators Assignment Areas

Four areas were considered for grouping the KPIs according to the complex interventions challenges [4] and the intervention strategy main characteristics, as shown in Fig. 2.

- In the change management area, the organization of local governance to plan, lead and coordinate the actions necessary to achieve change is required to activate a gradual, strategic, and responsible process. Therefore, the objective of this intervention strategy area items was to activate local teams, perform constant communication and dissemination activities, and deliver the necessary training for health teams. To achieve the minimum implementation of this section, the center must have managerial support, internal leadership for the installation of the model, and a local induction plan for the strategy for new employees. The measurement of these last three corresponds to the KPIs.
- In the operational area, it is necessary to perform modifications to the structure and health services delivery organization to allow the installation of the new care model. The objective was to assess the incorporation of multimorbidity stratification, changes in the protocols for electronic clinical records (ECR), and health services delivery according to each patient’s complexity. To achieve the minimum implementation of this section, the center must have the adult population stratification, unified drug prescriptions, alerts for consultations in the emergency service, and hospitalization activated and modify the acts on the agenda toward comprehensive care.
- Incorporating new roles is expected to provide new activities of the intervention strategy, such as Case Manager, Transition Nurse, Clinical Pharmaceutical Chemist, and High-Risk Family Physicians. The objective was to measure the degree of implementation of the new roles proposed to guarantee the execution of the new clinical services and improve continuity of care and patient follow-up efficiency. To achieve the minimum implementation of this section, the center must have the new roles installed.
- In the activities and services area, the differentiation of health care delivery by multimorbidity risk is a core aspect of the new care model and reflects the transition from a single diagnostic to a person-centered approach. The objective was to evaluate the core activities and services that would be the foundation for the sustainability of the change in healthcare delivery. To achieve the minimum implementation of this section, the center must have included the implementation of agreed plans, telephone counseling, continuity of care with a professional from the team, rescue after hospital discharge, implementation of an induction plan, and transition care.

### Key performance Identification

The objective was to identify components of the intervention strategy that were core for the change towards a multimorbidity approach and the implementation success. They were chosen based on the minimum conditions required for the intervention sustainability, on the representation of the implementation progress, on the availability of measurement information (either because it was available or because it was simple to download by the health team), and on accessible and sustainable monitoring over time.

From a total of 32 components, 17 were identified and assigned key performance indicators to track their implementation progress (Table 1).

**Table 1:** Indicators area and components

Area	Components
**Change Management**	Decision makers support (PHC director and managers)
	Leader for the implementation of the MPCM at the PHC
	Local training plan of MPCM for new employees
**Operational**	Adult population stratified by risk, available and with patients ID
	Unified drug prescription
	Alert system informing PHC teams of patients consulting at emergency room and hospitalization
	Integrated multimorbidity scheduled appointments
**New Roles**	Clinical Pharmacist
	High-Risk primary physician
	Case Manager
	Transition Nurse
**Activities and services**	Individualized Care Plans
	Phone counseling
	Continuity of care with a professional from the team
	Rescue after hospital discharge
	Implementation of an induction plan
	Transition care

PHC: Primary healthcare center

MPCM: Multimorbidity Patient-Centered Care Model

ID: identification number

### Complementary indicators identification

In addition to the KPIs, we developed another 15 indicators where the intervention strategy components open evaluation in greater depth if necessary. In the present study, we only evaluated the KPIs. The performance indicators for the MPCM are available in the supplementary material.

### Setup, measurement, and score assignment of KPI

The monitorization of the KPIs was self-reported, with dichotomous responses, and was completed by the implementation health care teams composed by clinicians such as nurses, physicians, nutritionist and physiotherapist. The setting-up, measurement, and scoring of KPI were provided by the study’s researchers and the expert team of CISAUC. The KPIs were designed according to each area and component. And a score was defined according to the level of complexity and relevance where the component was tracked.

For scoring, the individual scores of the KPI of each area were summed (example in change management score of 3) and divided by the maximum expected (score 28) to obtain a percentage of progress for each area (example: (3/28) * 100 = 10%). Finally, an average between areas was calculated for an overall percentage score. Table 2 represents the four groups of KPI scoring. The full description of scoring and measurement for each KPI is in the supplementary material.

**Table 2:** Key performance indicators areas

Area	Score (min-max)	Percentage of the total score (= group score/total score) (min-max)
Change Management	0–3	0–10%
Operational	0–9	0–29%
Incorporation of New Roles	0–4	0–13%
Activities and services	0–15	0–48%​
**Total Score**	**0–31**	**0-100%**

### Threshold and minimum implementation period

An overall threshold of 67% was defined with a group of experts and local teams to determine the minimal expected progress after 12 months of implementation in activities that are core to reflect the change. The implementation of MPCM represents a complex change, and the implementation of the complete intervention strategy is expected to be longer than the piloting period. Therefore, defining a minimal implementation period and a minimal percentage of implementation progress was relevant.

### Review with the primary care team

The KPIs’ setup, measurement, scoring, and pertinence were reviewed and discussed with the healthcare teams of the seven PHCs. Then a new draft was produced and checked for a second time to proceed and consolidate a final draft. The objective was to evaluate (i) the assertiveness of the KPIs with the minimal required conditions, (ii) the monitoring feasibility, and (iii)the understanding of a variety of healthcare professionals. Finally, the CISAUC team collected the information and made the necessary adjustments to the components and the indicators. This process was done twice, first after the indicators’ preliminary draft (December 2019). The second consisted in adapting the indicators to the global and national context of the COVID-19 pandemic (November 2020).

### Monitoring KPI

The seven PHCs had 30 days to monitor, collect the necessary information and fulfill the information of the indicators. This process was carried out in September 2020. During this period, the process was conducted by a local health care professional in charge of implementing the MPCM and supported by the CISAUC team. In addition, a document was prepared and delivered to the teams to facilitate the monitoring, collection, and completion of the information required and standardize the process.

The data with the results was collected and analyzed by the CISAUC expert team from each PHC that implemented the MPCM.

## Results

The intervened PHC were located at the southeast of the capital of Chile, Santiago and implemented the MPCM between 2017 and 2020. The population covered ranged from 17,487 to 35,240 patients. Three of the PHC were located at the municipality of La Pintana, two in La Florida and two in Puente Alto. The number of local care teams ranged from two to six for each PHC [12] (local team integrated by physician, midwives, nutritionists, physical therapists, psychologists, social workers, dentists, nurses and paramedic technician).

The overall results on the seven PHCs on 2020 showed positive implementation progress of the MPCM. The average total score was of 22 out of a maximum of 31. The overall threshold was met with a score of 72% (min 45% - max 100%) (Table 3).

**Table 3:** Results of the KPIs by area and component for each municipality and PHC.

Area	Component	Score	Municipality 1		Municipality 2			Municipality 3	
			PHC 1	PHC 2	PHC3	PHC4	PHC5	PHC 6	PHC 7
**Change Management**	Decision makers support (PHC director and managers)	1	1	1	1	1	1	1	1
	Leader for the implementation of the MPCM at the PHC	1	1	1	1	1	1	1	1
	Local training plan of MPCM for new employees	1	1	0	1	1	0	1	1
**Change Management Total Score**		3	3	2	3	3	2	3	3
**Operational**	Adult population stratified by risk, available and with patients ID	3	3	3	3	3	3	3	1
	Unified drug prescription	3	3	3	3	3	3	1	3
	Alert system informing PHC teams of patients consulting at emergency room and hospitalization	1	1	1	1	1	1	1	1
	Integrated multimorbidity scheduled appointments	2	2	1	2	2	0	2	2
**Operational Conditions Total Score**		9	9	8	9	9	7	7	7
**New Roles**	Clinical Pharmacist	1	1	1	1	1	1	0	0
	High-complexity primary physician	1	1	1	1	1	1	1	1
	Case Manager	1	1	1	1	1	1	1	1
	Transition Nurse	1	1	1	1	1	1	1	1
**New Roles total Score**		4	4	4	4	4	4	3	3
**Activities and services**	Individualized Care Plans	3	3	1	3	3	0	3	2
	Phone counseling	3	3	0	2	3	0	0	2
	Continuity of care with a professional from the team	3	0	0	0	3	0	0	2
	Rescue after hospital discharge	1	0	0	0	1	0	0	1
	Implementation of an induction plan	3	3	0	3	3	0	3	0
	Transition care	2	2	2	1	2	1	2	2
**Activities and services total Score**		15	11	3	9	15	1	8	9
**Total Score**		31	27	17	25	31	14	21	22
**Implementation progress percentage**		100%	87%	55%	81%	100%	45%	68%	71%

PHC: Primary healthcare center

MPCM: Multimorbidity Patient-Centered Care Model

ID: identification number

*The indicators description is available in supplementary material.

The municipalities that implemented the MPCM offered health services for similar populations showing differences in between. In Municipality 1, one of the PHCs obtained the highest level of implementation. On the contrary, the other PHC didn´t reach the minimum implementation threshold, scoring a 55% of implementation progress and lower results in activities and services. In Municipality 2 had similar results, where two of the three PHCs scored 81% and 100% on implementation progress, with high scores in components in the four areas. The third PHC didn’t reach the threshold and scored 45% in the implementation progress. Finally, in Municipality 3, both PHCs reached the threshold with scores of 68% and 71%.

Regarding the areas of evaluation, the highest scores were in change management and new roles. The lowest score was in services and activities. This is where the indicators reflect substantial changes in the real practice and the execution of the components of the intervention strategy of the MPCM.

The results by each component study showed that there were six that scored the highest: Decision makers support (PHC director and managers), Leaders for the Implementation of the MPCM at the PHC, Alert System Informing PHC Teams of Patients Consulting at the Emergency Room and Hospitalization, High-complexity Primary Physician, Case Manager, and Transition Nurse. In contrast, the components that obtained the lowest scores across all centers were: Continuity of Care and Rescue of High-risk Patients After Discharge.

Finally, regarding the review process with primary care teams, adjustments were provided in components mainly deciding if they were a “minimum or not” for the sustainability of the model. For example, in change management area the induction plan was a complementary indicator and after the review it was assessed as a KPI. In operational items, the Integrated multimorbidity scheduled appointments indicator was modified from a percentage of change to a dichotomic answer in yes/no. New roles had no modifications. Services and activities the Implementation of an induction plan and transition care were identified as KPI instead of complementary.

## Discussion

The results of the study showed that the MPCM intervention strategy can be monitored by the health care teams in terms of implementation progress through key performance indicators. Of the seven pilot centers, five (71,4%) reached the expected threshold reflecting the presence of the minimum intervention strategies required for the sustainability of the MPCM. Only two (28,6%) didn’t meet the threshold, demanding further attention to improve quality and performance. The results of this monitoring of the KPIs delivered relevant information for decision-makers and implementation teams to analyze and optimize the implementation progress.

Regarding the territory and the PHC where the MPCM pilot was implemented, the centers that did not reach the implementation progress threshold are from different municipalities but have in common the absence of important intervention strategy components. For example, the absence of integrated multimorbidity scheduled appointments refers to health professionals’ schedules by multimorbidity risk instead of by pathology or the program. Also, individualized plans and continuity of care were absent. These three missing components require a deeper and structural change in the organization and operation of the daily routine. Therefore, the barriers within the diagnostic approach for chronic [16–18] diseases are captured by the KPIs monitoring. Thus, strong decision-makers support is needed to authorize and facilitate the transition and sustainable change over the structural organization.

The areas of the KPIs also showed a relationship with the implementation process. The differences between the areas of progress may be related the stages of the pilot. In the pre-implementation phase, interventions were carried out first with a focus in a cultural and paradigm change, therefore, change management, operational, and new role changes began executed and obtained the highest scores. In contrast, the services and activities had less score reflecting that structural and operational chances diverse areas of the health services require a longer time [3]. Therefore, the importance to invest time and perform actions to properly install the basis of a further change [2]. Thus, a gradual process should be performed to ensure success in the implementation and sustainability overall as described in other studies. [2]

Concerning the areas, Change Management and New Roles reached the highest score. These results could be a consequence of the time invested in the pre-implementation period, where the action of socializing with the health teams, managers, and local leaders was frequent and essential to the change and its urgency. In addition, these changes don’t necessarily involve a structural change in the real context. Therefore, its implementation doesn’t face those barriers that are more difficult to address. Thus, the human resources inserted by the pilot study for the performance of the new roles was a challenge that had a positive acceptance from the health care teams, which probably positively influenced this area’s results [19]. Even more, the national scale-up of a similar intervention by the Ministry of Health included the new roles piloted [20].

The strength of the indicators is that they provide a simple, quantitative, and practical tool to monitor progress in multicomponent and interdisciplinary complex interventions. Methodologies described in the literature for health intervention design and implementation don’t usually include performance indicators or measures from the implementation process itself [3, 21]. Rather, they look for health outcomes [21, 22]. Therefore, complementing both could give health professionals and decision-makers a wider perspective with concrete gaps that certainly facilitate planning opportune quality improvement and addressing gaps in core areas to favor sustainability over time.

The limitations of the indicators are that they focus on the primary care components of the intervention strategy. Due to the piloting time, indicators for the performance in secondary and tertiary care were not provided but we included the most relevant network coordination activities performed, indicators such as transition care and rescue after hospital discharge measuring the continuity of care between care levels. Another limitation is that the second measurement was done in the first six months of the pandemic in Chile, which could have affected the results. Also, the validity of the indicators [23], therefore the construction of the KPI was reviewed, discussed twice with health care teams from the pilot centers. Finally, these are self-reported indicators, which could generate bias in their measurement as a proper limitation of the KPIs [5]. Hence, driving to an automatized monitoring could mitigate bias and maintain the strengths of a tool that delivers opportune, concrete, and relevant information for decision makers [24].

Finally, the set of key performance indicators has the potential to reflect the progress in a complex intervention in health like the MPCM, even though in a pandemic context. The automatization and extrapolation to other complex interventions in other groups of patients could provide early useful information to make opportune necessary changes and increase the expected outcomes of the intervention. The setup, monitoring and knowledge performed by the study it is potentially valuable for the similar intervention that it is scaling up the Ministry of Health [20]. Further studies could complement the indicators in the performance of the secondary and tertiary level providing a complete overview of the progress implementation of complex health interventions.

### Electronic supplementary material

Below is the link to the electronic supplementary material.

Supplementary Material 1

## Data Availability

All data generated or analyzed during this study are included in this published article and its supplementary information files.

## List of abbreviations

CISAUC: Centro de Innovacion en Salud ANCORA UC
ECR: Electronic clinical records
CESFAM: Family Health Center
KPI: Key Performance Indicator
MPCM: Multimorbidity Patient-Centered Care Model
FONASA: National Found of Health
PHC: Primary health care centers
SSMSO: Servicio Metropolitano Sur Oriente
