# Correction to: evaluation of the implementation progress through key performance indicators in a new multimorbidity patient-centered care model in Chile

**DOI:** 10.1186/s12913-023-09797-7

**Published:** 2023-07-12

**Authors:** Teresita Varela, Paula Zamorano, Paulina Muñoz, Carolina Rain, Esteban Irazoqui, Jaime C. Sapag, Alvaro Tellez

**Affiliations:** 1 Centro de Innovación en Salud ANCORA UC, Facultad de Medicina, Pontificia Universidad Católica de Chile, Santiago, Chile; 2 Health Technology Assessment Unit, Center of Clinical Research, Pontificia Universidad Católica de Chile, Santiago, Chile; 3 Present Address: Diagonal Paraguay, Santiago, 362 Chile; 4 Department of Family Medicine, Pontificia Universidad Católica de Chile, Santiago, Chile; 5 Department of Public health, Pontificia Universidad Católica de Chile, Santiago, Chile; 6 Dalla Lana School of Public Health, University of Toronto, Toronto, Canada

**Correction to**: *BMC Health Services Research* (2023) 23:439. 10.1186/s12913-023-09412-9.

Following publication of the original article [1], multiple errors (missing words) were identified throughout the main text, due to a typesetting mistake.

The original article [1] has been corrected and the publisher apologises to the authors and readers for the inconvenience caused by these errors.
