# Supplementary material for: Evaluation of the implementation progress through key performance indicators in a new multimorbidity patient-centered care model in Chile
Source: BMC Health Serv Res. 2023 May 4;23:439. doi: 10.1186/s12913-023-09412-9 (PMC10159678; doi:10.1186/s12913-023-09412-9)
Supplement: Supplementary file 1 — Supplementary Material 1 [file 12913_2023_9412_MOESM1_ESM.docx]

Supplementary Material

| **Area** | **Components** | **Key performance indicators** | **Maximum score** |  |
| --- | --- | --- | --- | --- |
|  |  |  |  |  |
| **Change Management** | Decision makers support (PHC director and managers) | YES = 1, NO= 0 | 1 |  |
|  | Leader for the implementation of the MPCM at the PHC | YES = 1, NO= 0 | 1 |  |
|  | Local training plan of MPCM for new employees | YES = 1, NO= 0 | 1 |  |
| **Operational** | Adult population stratified by risk, available and with patients ID | LOW RISK YES =1, NO=0; MODERATE RISK YES =1, NO=0; HIGH RISK YES =1, NO=0 | 3 |  |
|  | Unified drug prescription | LOW RISK YES =1, NO=0; MODERATE RISK YES =1, NO=0; HIGH RISK YES =1, NO=0 | 3 |  |
|  | Alert system informing PHC teams of patients consulting at emergency room and hospitalization | ONLY IN HIGH RISK: YES =1, NO=2 | 1 |  |
|  | Integrated multimorbidity scheduled appointments | LOW RISK AND MODERATE RISK YES =1, NO=0; HIGH RISK YES =1, NO=0 | 2 |  |
| **New Roles** | Clinical Pharmacist | YES = 1, NO= 0 | 1 |  |
|  | High-complexity primary physician | YES = 1, NO= 0 | 1 |  |
|  | Case Manager | YES = 1, NO= 0 | 1 |  |
|  | Transition Nurse | YES = 1, NO= 0 | 1 |  |
| **Activities and services** | Individualized Care Plans | (Nº of Individualized Care Plan made in the last control for high-risk patients´ / Total Nº of high-risk patients´ under control) ≥ 80%. YES = 2, NO= 0 | 3 |  |
|  |  | (Nº of Individualized Care Plans made in the previous control for medium and low-risk patients´ / Total number of selected medium and low-risk patients’ risk) ≥ 80%. YES = 1, NO= 0 |  |  |
|  | Phone Counseling | (Nº of high-risk patients with Phone Counseling performed/Nº of high-risk patients in case management) ≥ 95% (expected minimum 2 Phone Counseling per year per patient). YES = 2, NO= 0 | 3 |  |
|  |  | (Nº of medium and low-risk patients with Phone Counseling performed/Total Nº of selected medium and low-risk patients) ≥ 40% (expected minimum 1 Phone Counseling per year per patient). YES = 1, NO= 0 |  |  |
|  | Continuity of care with a professional from the team | (Nº of high-risk patients´ who have a primary care professional in the last two check-ups/Total Nº of high-risk patients) ≥ 90%. YES = 2, NO= 0 | 3 |  |
|  |  | (Nº of medium and low-risk patients´ who maintain their GP in the last two check-ups/Total Nº of selected medium and low risk patients´) ≥ 60%. YES = 1, NO= 0 |  |  |
|  | Rescue after hospital discharge | (Nº of patients´ contacted within 30 days after discharge from PHC in the last year/Total Nº of patients´ with hospital discharge in the last year) ≥90%. YES = 1, NO= 0 | 1 |  |
|  | Implementation of an induction plan | (N° of MPCM induction document delivery to new staff in the last three months/Total number of new staff in the last three months) ≥90%. YES = 1, NO= 0 | 1 |  |
|  | Transition care | (Nº of patients´ contacted by Transition Nurse within the first 7 days after discharge in the last year/ Total Nº of patients´ with hospital discharge in the last year who have been discharged for at least 7 days) ≥90%. YES = 1, NO= 0 | 1 |  |
|  |  | (Nº of patients´ contacted by Transition Nurse 30 days after discharge in the last year/ Total Nº of patients´ discharged from hospital in the last year) ≥90%. YES = 1, NO= 0 | 1 |  |
